# Improved accuracy of functional alignment restoration with robotic-assisted total knee arthroplasty: A quasi-randomized controlled trial

**DOI:** 10.1007/s00132-025-04711-x

**Published:** 2025-09-15

**Authors:** Filippo Migliorini, Luise Schäfer, Jens Schneider, Andrea Maria Nobili, Daniel Kämmer, Andreas Bell

**Affiliations:** 1 Department of Trauma and Reconstructive Surgery, University Hospital Halle, Martin-Luther University Halle-Wittenberg, Ernst-Grube-Street 40, 06097 Halle (Saale), Germany; 2 Department of Orthopaedic and Trauma Surgery, Academic Hospital of Bolzano (SABES-ASDAA), Via Lorenz Böhler 5, 39100 Bolzano, Italy; 3 Department of Life Sciences, Health, and Health Professions, Link Campus University, Via del Casale di San Pio V, 00165 Rome, Italy; 4 Department of Orthopaedic and Trauma Surgery, Eifelklinik St. Brigida, Kammerbruschstr. 8, 52152 Simmerath, Germany

**Keywords:** Robotic surgery, Joint line obliquity, Coronal plane, Valgus knee, Varus deformity, Roboterchirurgie, Joint line obliquity, Koronalebene, Valgusknie, Varusdeformität

## Abstract

**Background and objectives:**

This quasi-randomized controlled trial compared robotic-assisted and conventional total knee arthroplasty (TKA) in restoring patient-specific coronal alignment according to a functional alignment philosophy. Outcomes included preservation of the anatomical hip-knee-ankle angle (aHKA), mechanical axis deviation (MAD), and component positioning. Subgroup analyses assessed consistency in valgus and varus morphotypes.

**Material and methods:**

A single-blind parallel group quasi-RCT was conducted at the Department of Orthopedic Surgery, Eifelklinik St. Brigida, Germany (2021–2025). Patients underwent TKA following identical perioperative pathways. All procedures used a medial parapatellar approach and Smith & Nephew Legion Genesis II implants.

**Results:**

A total of 692 patients were enrolled (346 robotic, 346 freehand). Baseline characteristics were comparable. Postoperative HKA was similar between groups, but the robotic group achieved a significantly smaller aHKA delta (2.58° vs 4.49°, *p* = 0.002). Robotic-assisted TKA preserved joint line alignment more consistently in valgus (2.63° vs 5.72°, *p* = 0.03) and varus knees (2.56° vs 4.22°, *p* = 0.004). The MAD control was improved, while differences n the mechanical lateral distal femoral angle (mLDFA) and mechanical medial proximal tibial angle (mMPTA) were not significant.

**Conclusion:**

Robotic-assisted TKA enhanced accuracy in reproducing native joint line orientation, offering more consistent preservation of joint line obliquity in both valgus and varus morphotypes, thus supporting its role in personalized alignment strategies.

**Graphic abstract:**

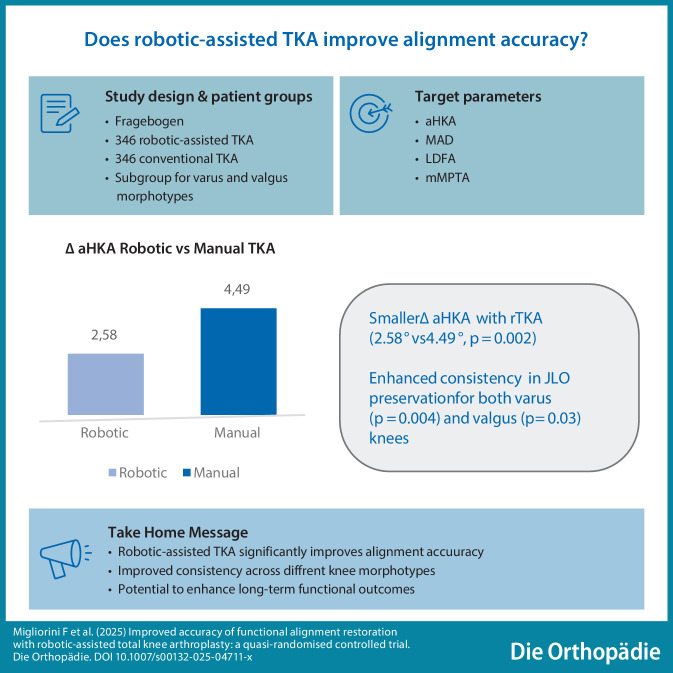

## Abstract (de)

**Hintergrund und Ziele:**

Diese quasi-randomisierte kontrollierte Studie verglich roboterassistierte und konventionelle Knietotalendoprothesen (TEP) hinsichtlich der Genauigkeit bei der Wiederherstellung der patientenspezifischen koronalen Ausrichtung nach funktionellem Alignment. Bewertet wurden der anatomische Hüft-Knie-Knöchel-Winkel (aHKA), die mechanische Achsabweichung (MAD) und die Komponentenpositionierung. Subgruppenanalysen untersuchten die Konsistenz bei Valgus- und Varus-Morphotypen.

**Material und Methoden:**

Es handelte sich um eine einfach verblindete Parallelgruppen-Quasi-RCT an der Eifelklinik St. Brigida, Deutschland (2021–2025). Alle Patienten erhielten einen medialen parapatellaren Zugang, identische perioperative Abläufe und Smith & Nephew Legion Genesis II Implantate.

**Ergebnisse:**

Insgesamt wurden 692 Patienten eingeschlossen (346 roboterassistiert, 346 konventionell). Ausgangscharakteristika waren vergleichbar. Der postoperative HKA-Winkel war in beiden Gruppen ähnlich, jedoch war das aHKA-Delta in der Robotergruppe signifikant kleiner (2,58° vs. 4,49°, *p* = 0,002). Roboterassistierte TEP zeigte konsistent bessere Ergebnisse bei Valgus- (2,63° vs. 5,72°, *p* = 0,03) und Varusknien (2,56° vs. 4,22°, *p* = 0,004). MAD wurde enger kontrolliert, Unterschiede bei mLDFA und mMPTA blieben ohne Signifikanz.

**Schlussfolgerung:**

Die roboterassistierte TEP zeigte eine höhere Genauigkeit bei der Reproduktion der Gelenklinienorientierung und verbesserte die Erhaltung der „joint line obliquity“ in Valgus- und Varusknien, was den Einsatz für präzise, individualisierte Alignment-Strategien unterstützt.

## Introduction

Knee osteoarthritis is common and negatively impacts the quality of life and activity levels of affected patients [1, 2]. In selected patients with end-stage knee osteoarthritis, total knee arthroplasty (TKA) may be necessary [3, 4]. The TKA is associated with high patient satisfaction; however, even in technically successful TKA [5, 6], confirmed by both clinical and radiographic assessments, some patients experience chronic postoperative pain. The causes of persistent pain following TKA are multifactorial, including component mispositioning or malalignment, patellofemoral disorders, and implant oversizing or overstuffing [7–9]. Enhancing surgical accuracy through more precise bone resections and optimizing component alignment may contribute to improved functional outcomes and patient satisfaction [6, 10, 11]. Under these premises, robotic-assisted TKA has developed [12, 13]. Evidence-based analyses demonstrate that robotic-assisted TKA might improve implant positioning accuracy compared to conventional freehand TKA, with robotic systems consistently reducing the incidence of malalignment and radiographic outliers [14–17]; however, to date, no published studies have directly compared the CORI robotic system (Smith & Nephew PLC, Watford, UK) with conventional freehand TKA regarding coronal alignment accuracy. This gap in the literature underscores the need for dedicated investigations to assess whether image-free robotic assistance provides enhanced radiographic precision in restoring patient-specific alignment targets.

The purpose of this quasi-randomized controlled trial (RCT) was to evaluate the accuracy of robotic-assisted TKA in restoring patient-specific coronal alignment based on a functional alignment philosophy, compared to conventional manual techniques. Specifically, the study assessed whether robotic surgery more effectively preserved the native anatomical hip-knee-ankle angle (HKA), mechanical axis deviation (MAD), and coronal component positioning (mechanical lateral distal femoral angle (mLDFA) and mechanical medial proximal tibial angle (mMPTA)). Subgroup analyses were conducted to determine the consistency of alignment restoration in both valgus and varus morphotypes. The aim was to ascertain whether robotic-assisted surgery provides superior precision in imaging to achieve personalized alignment targets in TKA compared to the standard freehand technique.

## Methods

### Study design

The present study is a single-blind, parallel-group, quasi-RCT, in which each group of participants is exposed to only one arm of the study interventions. The present clinical trial was performed according to the Consolidated Standards of Reporting Trials: the CONSORT statement [18]. All patients undergoing TKA at the Department of Orthopaedic Surgery of the Eifelklinik St. Brigida in Simmerath, Germany, between 2021 and 2025 were prospectively invited to participate in the present investigation. The present study was conducted in accordance with the principles of the Declaration of Helsinki and its subsequent amendments. The study protocol has been prospectively registered and approved by the German Register of Clinical Trials (ID DRKS00030614). Ethics approval has been received from the North Rhine Medical Council, Dusseldorf, Germany (ID 2022374). The protocol of the present quasi-RCT was previously published [19].

### Randomization and blinding

Patients who agreed to participate in the present study were informed preoperatively of the purpose of the research and signed a written informed consent form to confirm their willingness to participate in the trial. Patients were randomly allocated to robotic TKA or standard TKA. Randomization was performed in a 1:1 manner upon patient telephone contact, with each patient sequentially scheduled with either a surgeon using a robotic-assisted technique or one performing freehand TKA. Patients were blinded to the allocation until the first postoperative day (POD). Surgeons and personnel involved in the clinical management of the patients were unblinded to the allocation. Data collection and accuracy were conducted by two assessors who were blinded to group allocation and were not involved in the clinical management of the patients.

### Eligibility criteria

The inclusion criteria are: (1) age above 18 years, (2) capacity to consent, (3) symptomatic knee osteoarthritis stages II–IV according to the Kellgren-Lawrence classification [16]. The exclusion criteria are: (1) acute or chronic inflammatory diseases, (2) neoplastic diseases, (3) pregnancy and lactation, (4) uncontrolled coagulopathy, (5) abnormal cell count, (6) severe peripheral neuropathy, (7) vascular diseases, (8) peripheral ulcers, (9) other conditions that could influence the results of the present study.

### Outcomes of interest

On admission, the demographic information (age at surgery, body mass index, BMI, sex) was retrieved. The outcome of interest was to compare the implant positioning between robotic-assisted and conventional freehand TKA. Implant positioning was evaluated using anteroposterior plain radiographs of the lower leg with the software MediCAD Knie 2D (mediCAD Hectec GmbH, Altdorf, Germany). All radiographs were taken at POD5.

### Alignment targets

At our institution, we have adopted a functional alignment philosophy for TKA using the CORI system. This approach aims to replicate the patient-specific prearthritic anatomy and restore native biomechanics by preserving joint line orientation and soft tissue balance, rather than imposing a mechanically neutral alignment. The functional alignment model recognizes the natural variability of constitutional limb alignment in healthy individuals and seeks to reproduce the native coronal joint line obliquity (JLO) and the HKA within acceptable safety margins [20]. Our radiographic targets were carefully defined based on published recommendations for morphotype-specific alignment and stratified for varus and valgus deformities [21, 22]. We emphasized the restoration of the anatomical hip-knee-ankle angle (aHKA), as calculated by the difference between mMPTA and mLDFA. Maintenance of the native aHKA was prioritized to protect joint line inclination. Additionally, MAD was considered an independent radiographic outcome. The radiographic parameters are shown in Table 1.

**Table 1:** Alignment targets

Parameter	Varus morphotype target	Valgus morphotype target	Accepted accuracy threshold
HKA angle	174°–180° (up to 6° varus)	177°–183° (up to 3° valgus)	± 3° from morphotype-specific target
mLDFA	84°–96°	84°–96°	± 3°
mMPTA	84°–90°	85°–91°	Avoid valgus > 2° in valgus morphotype
aHKA	Maintain preoperative value	Maintain preoperative value	∆aHKA ≤ ± 3°
MAD	±10 mm from mechanical center	±10 mm from mechanical center	MAD within ±10 mm acceptable

The aHKA was considered as mMPTA-mLDFA

*HKA* hip-knee-ankle, *mLDFA* Mechanical Lateral Distal Femoral Angle, *mMPTA* Mechanical Medial Proximal Tibial Angle, *aHKA* anatomical HKA, *MAD* Mechanical Axis Deviation

### Surgical procedures and rehabilitation protocol

Surgeons who used robotic TKA had carried out at least 50 procedures before commencing this study. All patients adhered to the same clinical, imaging, and anaesthetic presurgical and postsurgical pathways, irrespective of their allocation. Each patient received a 1.5 g single dose of intravenous cefuroxime at the induction of anesthesia. A femoral nerve block was employed for pain control and maintained for 48 h. All surgeries were conducted using a standard medial parapatellar approach. All components were implanted according to the manufacturer’s instructions using the Smith & Nephew Legion Genesis II, featuring a posterior-stabilized polyethylene liner insert. Both femoral and tibial implants were cemented with Palacos cement (Heraeus Medical GmbH, Wehrheim, Germany). For the first 48 h, one closed suction deep drain and one open suction subcutaneous drain were used. Antithrombotic prophylaxis with enoxaparin sodium (40 mg/0.4 ml daily, subcutaneously) for 6 weeks, commenced 12 h after the index procedure. Physiotherapy followed standard protocols [21]. A team of physiotherapists followed patients during hospitalization from the first POD. In the absence of complications or other medical reasons that prevent discharge, the minimum length of hospitalization at our institution was 5 days. Moreover, from POD2, each patient underwent two daily sessions of physiotherapy, each lasting 60 min, using continuous passive motion (CPM) to flex and extend the knee joint. The physiotherapist increased the range of motion (ROM) at each session. Patients were discharged when they had reached at least 80° of flexion. Starting from POD2, patients began walking under physiotherapist supervision, and on POD4, they started ascending and descending stairs. A personalized outpatient or inpatient rehabilitation program was designed for every patient, lasting a minimum of 3 weeks. Deviation from the planned surgical procedure and rehabilitation protocol warranted exclusion from the study.

### Statistical analysis

Normality of continuous variables was assessed through the Shapiro-Wilk test. Based on distribution characteristics, non-parametric tests were adopted as the primary analytic strategy. Descriptive statistics included mean, standard deviation (SD), standard error (SE), and 95% confidence intervals (CI). Group comparisons were performed using the Mann-Whitney U test for non-normally distributed data and Welch’s t‑test for data with unequal variances. A two-tailed *P*-value < 0.05 was considered statistically significant. Exploratory comparisons included individual radiographic angles (mLDFA, mMPTA) and MAD. Subgroup analyses were performed for both valgus and varus morphotypes. Effect sizes and trends were interpreted in conjunction with CI to assess both statistical and clinical significance. A post hoc power analysis was performed to identify whether the sample size was adequate to support the results.

## Results

### Recruitment process

**Fig. 1:**
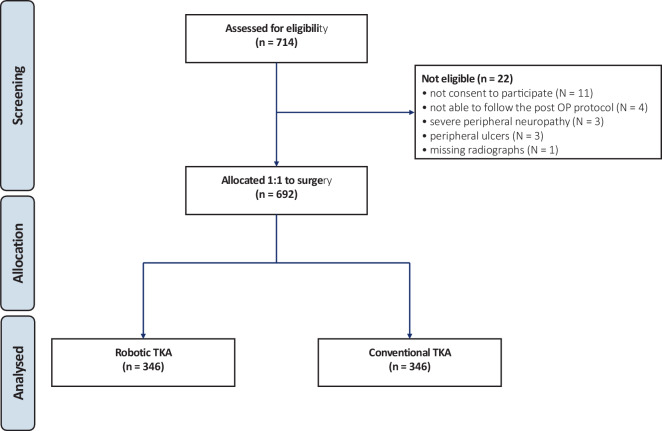
CONSORT diagram of the recruitment process

A total of 714 patients were initially recruited. Of these, 22 were deemed ineligible: lack of consent to participate (*N* = 11), inability to follow the postoperative protocol (*N* = 4), peripheral ulcers (*N* = 3), severe peripheral neuropathy (*N* = 3) and missing radiographs (*N* = 1). Ultimately, 692 patients underwent surgery: 346 were allocated to robotic TKA and 346 to conventional freehand TKA (Fig. 1).

### Patient demographics

The robotic TKA evidenced a statistically significant longer surgical duration (*p* = 0.04). No other difference was found in age, gender distribution, BMI, or preoperative limb alignment at admission (Table 2).

**Table 2:** Demographic data of the patients

Variable	Robotic group (*N* = 346)	Manual group (*N* = 346)	*P*
Age, mean ± SD (*years)*	69.6 ± 7.5	69.9 ± 7.8	0.6
Surgical duration *(mins)*	83.4 ± 17.8	80.5 ± 17.4	0.04
Women, *n* (*%*)	217 (62.7)	212 (61.3)	0.7
BMI, mean ± SD (*kg/m*^*2*^*)*	29.4 ± 3.6	29.7 ± 3.9	0.3
Preoperative HKA (°), mean ± SD	175.1 ± 4.8	174.8 ± 5.1	0.5
Valgus knees, *n* (*%*)	83 (24.0)	79 (22.8)	0.7
Varus knees, *n* (*%*)	263 (76.0)	267 (77.2)	0.7

*SD* Standard Deviation, *BMI* Body Mass Index, *HKA* Hip-Knee-Ankle

### Outcomes of interest

Postoperative HKA angle was comparable between the two groups, with the robotic group achieving a mean of 179.96° (SD 1.59, SE 0.086, 95% CI 179.80° to 180.12°) and the freehand group 179.88° (SD 3.62, SE 0.194, 95% CI 179.50° to 180.25°); (*p* = 0.5). In contrast, the absolute delta of aHKA was statistically significantly (*p* = 0.002) smaller in the robotic group (mean 2.58°, SD 2.46, SE 0.133, 95% CI 2.32° to 2.83°) than in the freehand group (mean 4.49°, SD 2.95, SE 1.59, 95% CI 1.37° to 7.61°).

When analyzing only valgus morphotypes, robotic-assisted TKA showed improved consistency in preserving joint line alignment, with a mean aHKA delta of 2.63° (SD 2.21, SE 0.163, 95% CI 2.31° to 2.95°) compared to 5.72° (SD 4.22, SE 3.58, 95% CI −1.32° to 12.75°) in the freehand group (*p* = 0.03). In varus knees, the robotic group demonstrated a mean aHKA delta of 2.56° (SD 2.51, SE 0.162, 95% CI 2.24° to 2.89°) while the freehand group exhibited a delta of 4.22° (SD 2.79, SE 1.71, 95% CI 0.87° to 7.56°); (*p* = 0.004).

The robotic group also showed tighter control in MAD and joint line orientation, although differences in mLDFA and mMPTA did not reach statistical significance. Exploratory comparisons of component angles and axis deviation yielded the following nonsignificant trends. These results are schematically reported in Table 3.

**Table 3:** Main results

Parameter	Robotic (*N* = 346)	Manual (*N* = 346)	*P*
HKA (°)	179.96 ± 1.59	179.88 ± 3.62	0.5
*∆* aHKA (°)	2.58 ± 2.46	4.49 ± 2.95	0.002
mLDFA (°)	89.1 ± 2.4	88.6 ± 3.0	0.2
mMPTA (°)	89.3 ± 2.5	88.2 ± 2.8	0.1
MAD (mm)	6.1 ± 5.1	6.7 ± 5.5	0.4
*Subgroup: valgus*	*Robotic* *(N* *=* *83)*	*Manual* *(N* *=* *79)*	*P*
∆ aHKA (°)	2.63 ± 2.21	5.72 ± 4.22	0.03
*Subgroup: varus*	*Robotic* *(N* *=* *263)*	*Manual* *(N* *=* *267)*	*P*
∆ aHKA (°)	2.56 ± 2.51	4.22 ± 2.79	0.004

*HKA* Hip-Knee-Ankle, *mLDFA* Mechanical Lateral Distal Femoral Angle, *mMPTA* Mechanical Medial Proximal Tibial Angle *MAD* Mechanical Axis Deviation, *aHKA* Anatomical Hip-Knee-Ankle

### Post hoc power analysis

Assuming a moderate effect size (Cohen’s d = 0.35), a two-tailed alpha level of 0.05, and equal group allocation, the required sample size to achieve 80% power was calculated to be 129 patients per group.

## Discussion

According to the main findings of the present RCT, robotic-assisted TKA demonstrated superior accuracy in reproducing the native joint line orientation. While global alignment measures, such as HKA, were equivalent between groups, the robotic technique significantly improved the preservation of JLO in both valgus and varus knees. These findings support the use of robotic technology in facilitating precise and personalized alignment strategies in TKA.

The radiographic parameters selected in this study were chosen for their relevance in evaluating coronal alignment and joint line preservation in TKA. Unlike neutral mechanical alignment, which imposes uniform correction regardless of individual anatomy, the functional alignment philosophy aims to replicate the native alignment and JLO specific to each patient’s constitutional varus or valgus morphotype [20, 23]. The clinical relevance of this quasi-RCT lies in its ability to demonstrate the superior accuracy of robotic-assisted TKA using the CORI imageless system in restoring patient-specific coronal alignment within a functional alignment philosophy. Unlike prior studies that have primarily focused on global alignment metrics, such as MAD or mechanical HKA angle [24, 25], the present study introduces a more anatomically meaningful analysis by emphasizing the restoration of the aHKA as a surrogate for JLO, which has a direct impact on native biomechanics and soft tissue tension. The aHKA has emerged as a clinically meaningful metric in TKA as it reflects the native joint line orientation and individual morphotype, making it particularly suitable for functional alignment strategies [26]. Unlike the mechanical HKA, which assumes a universal neutral target, the aHKA captures patient-specific coronal alignment and better correlates with native joint kinematics and soft tissue balance [27, 28]. Recent studies have validated the reliability and reproducibility of aHKA as a surrogate for JLO, demonstrating its utility in surgical planning and outcome prediction [29, 30]. Previous investigations involving robotic TKA have largely neglected morphotype-stratified outcomes or relied on image-based planning, which limits generalizability and increases resource burden [31, 32]. Moreover, none have specifically assessed the CORI image-free system within a functional alignment protocol using quantitative delta aHKA analysis. Clinically, the results of the present study underscore the potential of robotic-assisted TKA to minimize alignment outliers and preserve joint line orientation, which may lead to improved ligament balancing, reduced iatrogenic soft tissue tension, and ultimately a lower incidence of persistent postoperative pain despite otherwise perfect radiographic results.

Robotic-assisted TKA offers superior intraoperative control over bone resections and implant positioning, thanks to real-time feedback, preoperative planning integration, and millimeter precision [33]. This enhanced control is particularly valuable when attempting to reproduce morphotype-specific targets and to avoid alignment outliers, which may compromise kinematic performance and soft tissue balance [33, 34]. The use of robotic guidance was thus hypothesized to reduce alignment deviations, particularly in complex morphotypes, such as valgus knees, and better preserve aHKA and MAD within predefined safe zones [34]. This methodology aligns with recent evidence advocating for personalized, anatomy-respecting alignment strategies that optimize functional outcomes without compromising implant longevity [35].

The CORI system is a handheld robotic-assisted platform designed to enhance intraoperative precision in TKA [36]. Unlike image-based systems, which rely on preoperative imaging, CORI is an image-free registration workflow that eliminates the need for preoperative imaging, reduces radiation exposure, lowers costs, and streamlines surgical logistics [36]. This platform combines real-time optical tracking with intraoperative mapping of the femoral and tibial surfaces to generate a dynamic 3D model of the joint [36, 37]. It also enables evaluation and iterative adjustment of component positioning in response to ligament laxity and mechanical axis deviations before any bone resection is performed [36, 38]; however, despite these advantages, it remains uncertain whether this approach translates into superior alignment accuracy when compared to conventional freehand TKA. Previous studies have evaluated the accuracy of the CORI image-free robotic system in TKA [39]. An RCT [40] reported that CORI achieved a mean femoral component rotational alignment error of 1.33°, compared to 3.15° with conventional manual TKA (*p* = 0.001), aligning with similarly precise results from NAVIO (1.48°), the precursor of CORI in TKA navigation.

Computer-assisted knee arthroplasty was developed to enhance implant positioning, limb alignment, and surgical reproducibility by integrating intraoperative navigation systems [41, 42]. Traditional navigated systems provide real-time visual guidance based on anatomical landmarks and predefined axes, improving mechanical alignment without altering the surgical instrumentation itself [43]; however, these systems do not physically constrain the surgeon’s actions and rely on manual execution, thus leaving room for variability [44]. Robotic systems, in contrast, combine navigation with active or semi-active guidance, enabling controlled bone resections based on virtual planning [43, 45]. The NAVIO (navigation system) and CORI (robotic system), both developed by Smith & Nephew, represent successive generations of image-free handheld robotic platforms. The NAVIO requires extensive registration and probe-tracked mapping of bony landmarks and joint surfaces to build an intraoperative model [46]. The CORI, its evolution, maintains an imageless workflow but features enhanced optical tracking, automated registration, and an improved user interface, significantly reducing set-up time and allowing more refined planning and execution [36]. Technically, CORI uses a handheld sculpting tool equipped with haptic feedback and real-time tracking, enabling the precise execution of predefined resection planes with millimeter accuracy [36]. Unlike large console robotic systems, such as MAKO or ROSA, CORI does not require preoperative CT scans or cumbersome machinery, offering logistical advantages and reducing costs while preserving accuracy [40]. These differences make CORI particularly suited for settings prioritizing intraoperative flexibility and workflow integration.

The present study is a quasi-RCT in which patients were allocated 1:1 to a group based on the chronological order of their reservation. This modality might introduce a risk of selection bias and should be considered when interpreting the results. While the quasi-randomized allocation method may raise concerns regarding potential selection bias, the considerable sample size, far exceeding the calculated requirement, serves to counterbalance this limitation by increasing statistical power, reducing the risk of type II error and reinforcing the generalizability of the findings. A post hoc power analysis was conducted to determine whether the sample size of 346 patients per group was sufficient to detect clinically meaningful differences in radiographic alignment outcomes. Surgeon-related variability has long been recognized as a potential confounder in comparative studies on surgical technology, particularly in procedures such as total knee arthroplasty, where alignment accuracy and intraoperative decision-making play critical roles [47, 48]. In the present study, this source of bias was minimized by restricting all procedures to four high-volume senior surgeons, each with experience exceeding 1000 TKA cases. The same implant type, surgical approach, perioperative care protocol, and rehabilitation pathway were uniformly applied across both groups. Furthermore, the robotic and conventional procedures were performed by distinct but equally experienced teams, ensuring parity in surgical expertise between the two study arms. The lack of clinical outcome measures, such as pain scores, functional assessments, or patient-reported outcome measures, is another limitation of the present study. While the primary aim was to evaluate the accuracy of alignment and joint line restoration using robotic-assisted versus conventional TKA, clinical outcomes remain essential to understanding the translational relevance of radiographic precision; however, as predefined in the published study protocol, the evaluation of clinical and functional outcomes has been planned from the 2‑year follow-up onwards, in accordance with established evidence indicating that early postoperative assessments may not reliably reflect long-term recovery or patient satisfaction. The study revealed that the required sample size to achieve 80% power was calculated to be 129 patients per group. Therefore, the actual cohort size of 346 patients per arm provides a power greater than 99.5%, far exceeding the minimum threshold needed to detect moderate group differences. This substantial sample size enhances the robustness of subgroup analyses and enables more precise estimation of effect sizes and confidence intervals, while minimizing the risk of type II error. The use of a larger cohort also reflects real-world heterogeneity and supports the external validity of the study findings.

## Conclusion

Robotic-assisted TKA demonstrated superior accuracy in reproducing the native joint line orientation. While global alignment measures, such as HKA, were equivalent between groups, the robotic technique significantly improved the preservation of JLO in both valgus and varus knees. These findings support the use of robotic technology in facilitating precise and personalized alignment strategies in TKA.

## Data Availability

The datasets generated during and/or analyzed during the current study are available throughout the manuscript.

## Abbreviations

aHKA: Hip-knee-ankle angle
CI: Confidence intervals
CPM: Continuous passive motion
JLO: Joint line obliquity
MAD: Mechanical axis deviation
mLDFA: Mechanical lateral distal femoral angle
mMPTA: Mechanical medial proximal tibial angle
POD: Post-operative day
RCT: Randomised controlled trial
ROM: Range of motion
SD: Standard deviation
SE: Standard error
TKA: Total knee arthroplasty
